# Retroviral foamy virus gag induces parkin-dependent mitophagy

**DOI:** 10.1186/s12977-025-00664-3

**Published:** 2025-05-02

**Authors:** Shanshan Wang, Tongtong Du, Jun Yan, Yingcheng Zheng, Yinglian Tang, Juejie Wu, Qian Xu, Shanshan Xu, Luo Liu, Xiong Chen, Song Han, Jun Yin, Biwen Peng, Xiaohua He, Wanhong Liu

**Affiliations:** 1 Hubei Province Key Laboratory of Allergy and Immunology, Taikang Medical School (School of Basic Medical Sciences), Wuhan University, Wuhan, 430071 China; 2 Department of Laboratory Medicine, Wuhan Children’s Hospital, Tongji Medical College, Huazhong University of Science and Technology, Wuhan, 430071 China; 3 Department of Allergy, Zhongnan Hospital of Wuhan, University, Wuhan, 430071 China; 4 Beijing Bioprocess Key Laboratory, Beijing University of Chemical Technology, Beijing, 100029 China; 5 Key Laboratory of Environmental Pollution Monitoring and Disease Control(Guizhou Medical University), Ministry of Education, Guiyang, 550025 China

**Keywords:** Prototype foamy virus, Gag, Mitophagy, Parkin, Rab5a

## Abstract

**Background:**

Prototype foamy virus (PFV) is a complex retrovirus that can maintain latent infection for life after viral infection of the host. However, the mechanism of latent infection with PFV remains unclear. Our previous studies have shown that PFV promotes autophagy flux, but whether PFV causes mitophagy remains unclear.

**Results:**

In this study, we demonstrated that PFV infection damages mitochondria, increases mitochondria reactive oxygen species (mtROS) production, and induces mitophagy in a time-dependent manner. Further investigation revealed that PFV Gag is a crucial protein responsible for triggering mitophagy. The overexpression of Gag leads to mitochondrial damage and stimulates mitophagy in a dose-dependent manner. Additionally, overexpression of Gag activates the PINK1-Parkin signaling pathway, while the knockdown of Parkin inhibits Gag-induced mitophagy. Furthermore, Rab5a was significantly upregulated in cells overexpressed Gag, and the inhibition of Rab5a reversed the effects of Gag-induced mitophagy.

**Conclusions:**

Our data suggested that PFV can induce mitophagy and Gag induces Parkin-dependent mitophagy by upregulating Rab5a. These findings not only enhance a better understanding of the foamy virus infection mechanisms but also provide critical insights into novel virus-host cell interactions.

**Supplementary Information:**

The online version contains supplementary material available at 10.1186/s12977-025-00664-3.

## Introduction

Foamy virus is the oldest known retrovirus [1], belonging to the spumaretrovirinae subfamily within theretroviridae family. It is a single-stranded, positive-sense RNA virus. Human foamy virus was first isolated from nasopharyngeal cancer patients in the 1970s [2] and is referred to as the prototype foamy virus (PFV) due to its high homology with simian foamy virus (SFV) found in chimpanzees [3]. The PFV genome comprises *Gag*, *Env*, *Pol* which encode structural proteins, and other regulatory factors [4]. Notably, the Gag protein plays a crucial role in various processes of PFV replication. Upon infection, PFV establishes a latent infection that can persist for extended periods. Programmed cell death is a distinctive biochemical pathway that serves as a defense mechanism against various infections and diseases [5]. It has been found that the regulatory pathways of cell death mainly include apoptosis, necroptosis and autophagy [6]. In addition, it has been reported that Tas of SFVagm and SFVora (simian foamy viruses from Old World monkeys) activate mitochondria-mediated apoptosis pathway to inhibit cell proliferation [7]. However, the mechanism by which PFV induces cell death remains to be elucidated.

Mitophagy is a specific form of autophagy that selectively removes damaged mitochondria and plays a crucial role in maintaining mitochondrial homeostasis [8]. It is activated when mitochondrial damage surpasses the capacity of quality and quantity control, or when mitochondria are eliminated through cellular metabolism [9]. Recent studies have explored the role of mitophagy in various viral infections. Viruses have evolved diverse strategies to induce mitophagy for their own advantage [10]. Some viruses trigger intracellular events that lead to mitochondrial dysfunction, promoting either Parkin-dependent or Parkin-independent mitophagy in the host. Parkin-dependent mitophagy is typically influenced by the mitochondrial membrane potential (Δψm) [11]. Under hypoxic conditions or when invaded by external pathogens, mitochondrial depolarization activates the serine/threonine protein kinase PTEN-induced putative kinase 1 (PINK1), which detects mitochondrial damage and subsequently activates the E3 ubiquitin ligase Parkin in the cytoplasm. Parkin accumulates on the outer membrane of damaged mitochondria, undergoes phosphorylation, and mediates the formation of autophagosomes that engulf the impaired mitochondria, facilitating their degradation [12]. Parkin-independent mitophagy is mediated by receptors. Identified receptors that regulate mitophagy include Atg32, NIP3-like protein X (NIX), FUN14 domain-containing protein 1 (FUNDC1), and Bcl-2/adenovirus E1B 19 kDa interacting protein 3 (BNIP3), among others [13, 14]. When mitochondria are damaged, the autophagy receptors located on their outer membrane bind to autophagy-related proteins, enabling autophagosome to directly engulf the damaged mitochondria, which are subsequently cleared via autophagolysosome, thus maintaining mitochondrial homeostasis [15]. In the cases of hepatitis B virus (HBV) [16], hepatitis C virus (HCV) [17], venezuelan equine encephalitis virus (VEEV) [18], classical swine fever virus (CSFV) [19], porcine reproductive and respiratory syndrome virus (PRRSV) [20], newcastle disease virus (NDV) [21], and transmissible gastroenteritis virus (TGEV) [22] have been shown to induce mitophagy through various mechanisms, thereby affecting viral infection via this process. However, the role of PFV in regulating mitophagy and the underlying molecular mechanisms remain unclear.

Our previous studies have indicated that PFV can induce autophagy [23], and that the PFV Gag protein can promote endosome-related autophagy [24]. However, whether PFV induces mitophagy and the role of Gag in this process remain unclear. The dependence of long-term latent PFV infection on mitophagy is not yet fully understood. In this study, we demonstrate that PFV infection induces mitochondrial damage and triggers mitophagy, with the PFV Gag protein playing a crucial role in this process. Further investigations reveal that the PFV Gag protein promotes Parkin-dependent mitophagy by upregulating Rab5a. Our findings uncover a significant mechanism through which PFV induces mitophagy and provide insights into a novel pathway for mitochondrial quality control.

## Materials and methods

### Cells, virus and plasmids

HEK293T(ATCC, CRL-11268) cells were grown in Dulbecco modified Eagle medium (DMEM, HyClone) supplemented with 10% heat-inactivated fetal bovine serum (FBS, Biological Industries), HT1080 cells (ATCC, CCL-121) were maintained in minimum Eagle medium (MEM, HyClone) containing 10% FBS. Cells were incubated at 37 °C in a humidified atmosphere containing 5% CO₂.

The proviral plasmid pHSRV13 was transfected into HEK293T cells using the PEI transfection reagent. After transfection for 48 h, the cells and culture medium were repeatedly freeze-thawed three times to release the virus, centrifuged at 4 ℃ and 12,000 rpm for 15 min, and then the supernatant was taken and filtered with 0.22 μm filter membrane. HT1080 cells were infected with the viral supernatant for 2 h, the process was repeated to obtain higher viral titers.

The pHSRV13 provirus DNA was generously provided by Professor Rolf M. Flügel (German Cancer Research Center). PFV Gag genes were cloned into pcDNA6.0-His. shDNAs encoding specific shRNAs targeting Rab5a, Park2, and Atg5 were separately cloned into pLKO.1. pEnCMV-Rab5a-HA and GFP-mCherry-LC3 plasmids were purchased from MiaoLing biology.

### Reagents and antibodies

Anti-p62/SQSTM1, anti-Atg5, anti-Cox4, anti-Rab5a, and anti-β-actin antibodies were purchased from Proteintech. Anti-LC3B, anti-PINK1, and anti-Parkin antibodies were purchased from Cell Signaling Technology. Anti-Tom20 antibody was purchased from Abcam. HRP-conjugated goat anti-rabbit or goat anti-mouse secondary antibodies were purchased from Bioprimacy. Dylight 594-conjugated goat anti-mouse secondary antibody, Dylight 594-conjugated goat anti-rabbit secondary antibody, Dylight 488-conjugated goat anti-mouse secondary antibody, Dylight 488-conjugated goat anti-rabbit secondary antibody, and Dylight 405-conjugated goat anti-rabbit secondary antibody were purchased from Abbkine.

### RNA extraction and qPCR

Total RNA was extracted from cells with TRIzol reagent (Invitrogen). The RNA was reverse transcribed to cDNA using the HiScript III RT SuperMix for qPCR (Vazyme) according to the manufacturer’s instructions. Real-time qPCR was carried out using an CFX384 sequence detection system (Bio-Rad, USA). β-actin was used as an invariant control. Relative mRNA expression was evaluated using 2^−ΔΔCt^ method. The sequence of the gene primers used for qPCR are listed in Table 1.

**Table 1:** Primer sequences for each target gene

ID	Primer Sequence
Cox1 Forward primer	AACAGACCGCAACCTCAACA
Cox1 Reverse primer	AATAGGATGGTCCGAAGCCT
18 S rRNA Forward primer	ATCAAGAACGAAAGTCGGAGGT
18 S rRNA Reverse primer	CCCACGGAATCGAGAAAGAG
Rab5a Forward primer	AAGACCCAACGGGCCAAATA
Rab5a Reverse primer	GGTTAGAAAAGCAGCCCCAATG
β-actin Forward primer	CACGATGGAGGGGCCGGACTCATC
β-actin Reverse primer	TAAAGACCTCTATGCCAACACAGT
Gag Forward primer	AATAGCGGGCGGGGACGACA
Gag Reverse primer	ATTGCCACGCACCCCAGAGC
IFNB Forward primer	AACTGCAACCTTTCGAAGCC
IFNB Reverse primer	AGAAGCACAACAGGAGAGCA
CCL5 Forward primer	TCGTCCACAGGTCAAGGATGC
CCL5 Reverse primer	TTCGGGTGACAAAGCTTGCCC
CXCL10 Forward primer	AAGTGGCATTCAAGGAGTACCT
CXCL10 Reverse primer	GGACAAAATTGGCTTGCAGGA

### Mitochondrial membrane potential and mitochondrial ROS

According to the manufacturer’s instructions, the cells were incubated at 37 ℃ for 30 min with TMRM or MitoSOX. After incubation, the TMRM or MitoSOX was discarded, and the cells were washed three times with Hank’s buffer. The fluorescence of TMRM and MitoSOX was detected by flow cytometry(Beckman, USA)and analyzed quantitatively.

### Confocal microscopy

Cells were plated in confocal culture dishes (BioSorfa), transfected 24 h later, washed three times with PBS, and then fixed with 4% paraformaldehyde (BioSharp) at room temperature for 30 min. After 20 min of permeabilization with 0.1% Triton X-100 (Epizyme), the cells were blocked with 5% BSA for one hour, and then incubated with designated primary antibody, secondary antibody, and DAPI. The stained cells were observed by confocal microscope (Leica, Germany), with random fields selected for photography.

### Western blotting

After washing the cells three times with PBS, RIPA lysate containing PMSF was added and incubated on ice for 30 min. The cell lysate was then centrifuged at 4 ℃ and 12,000 rpm for 15 min. BCA protein assay kit (BioSharp) was used to detect the protein concentration. Samples were prepared by adding 5×loading buffer (BioSharp) and boiling for 15 min. The protein was separated with 10% or 12% SDS-PAGE and then transferred to PVDF membranes (Roche). After two hours of blocking with 5% milk, the specific primary antibodies were added and incubated in a 4 ℃ shaker overnight, and then incubated with the corresponding HRP-conjugated secondary antibodies at room temperature for two hours. The protein bands were visualized with ECL kit (BioSharp), and the gray values were measured with Image J software.

### Mitochondria extraction

After cell collection, mitochondria were isolated using a mitochondrial extraction kit (Solarbio) following the manufacturer’s protocol.

### Data analysis

Data are represented by Mean ± SEM. Comparisons between two groups were performed using Student’s *t*-tests. One-way ANOVA was used to analyze single-factor variables across more than two groups. Multivariate variables across two or more groups were analyzed using two-way ANOVA. *p* < 0.05 was considered statistically significant.

## Results

### PFV infection induces mitophagy

**Fig. 1:**
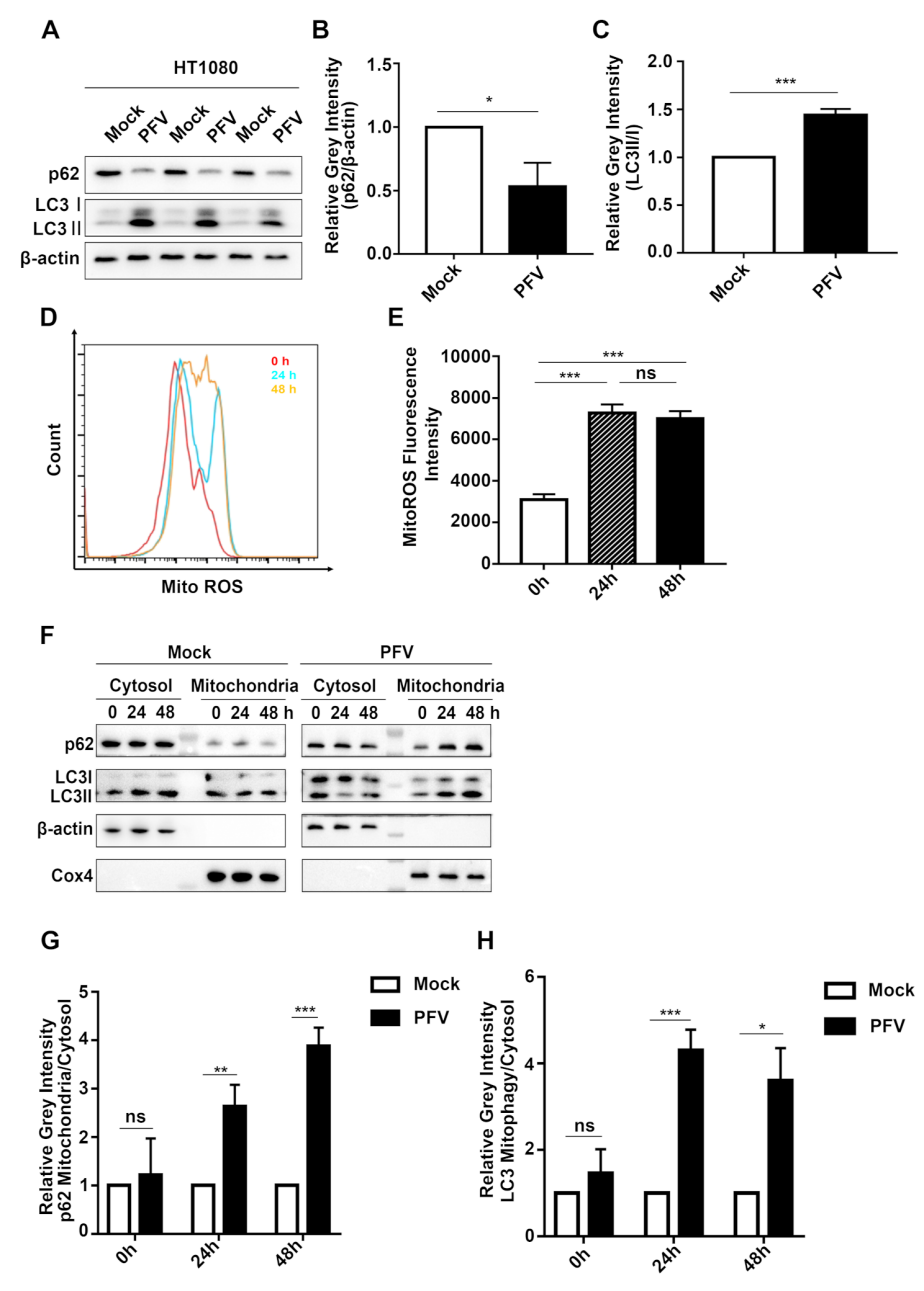
**(A)** HT1080 cells were treated with Mock or PFV (MOI = 0.5) for 48 h. Western blotting was performed to assess changes in the levels of p62 and LC3 II/I proteins. **(B-C)** Statistical analysis of the relative gray intensity of p62 and LC3 II/I was conducted, using Student’s *t*-test (*n* = 3, ***p < 0.001, *p < 0.05). **(D)** Following PFV infection of HT1080 cells at various time points (0 h, 24 h, 48 h), cells were incubated with 5 µM MitoSOX, protected from light, for 10 min. Fluorescence intensity changes were quantitatively analyzed using flow cytometry. **(E)** Statistical analysis of MitoSOX ROS fluorescence intensity is shown (*n* = 4, ***p < 0.001, ns *p* > 0.05). **(F)** After PFV infection of HT1080 cells for 0 h, 24 h, and 48 h, mitochondrial isolation was performed, and Western blotting was used to evaluate changes in p62 and LC3 II/I protein levels in both cytoplasmic and mitochondrial fractions. **(G-H)** The ratios of p62 and LC3 II/I protein content in the cytoplasm versus their respective content in mitochondria were statistically analyzed (Student’s *t*-test, *n* = 3, ***p < 0.001, **p < 0.01, *p < 0.05)

Our previous study have revealed PFV infection induces autophagy [24]. After infecting its susceptible cell line HT1080 with PFV for 48 h, we found that PFV infection promoted LC3 lipidation (Fig. 1A, C, ***p < 0.001) and p62 degradation (Fig. 1A, B, *p < 0.05) compared to Mock, which indicate that PFV infection indeed promotes autophagy. Mitochondria reactive oxygen species (mtROS) are involved in regulating immune response and autophagy related signaling pathways [25]. Mitochondrial damage leads to the increase of mtROS. Next, we explored whether PFV infection can lead to mitochondrial damage. We examined mtROS levels in HT1080 cells post-infection. As predicted, mtROS increased significantly at both 24 and 48 h after PFV infection (Fig. 1D, E, ***p < 0.001). Regards that damaged mitochondria may promote p62-mediated autophagic clearance (mitophagy), we reasoned that PFV might induce mitophagy. Since mitophagy leads to an increase in the mitochondrial-to-cytosolic ratios of LC3 and p62, we infected HT1080 cells with PFV at different time points, extracted mitochondria after cell collection, and detected the changes of p62 and LC3 protein levels in cytoplasm and mitochondria by western blot. Compared to the Mock group, the mitochondrial-to-cytosolic ratios of p62 (Fig. 1F, G, **p < 0.01) and LC3 (Fig. 1F, H, ***p < 0.001) were significantly increased in PFV-infected cells at 24 h. At 48 h post-infection, the mitochondrial-to-cytosolic ratios of p62 (Fig. 1F, G, ***p < 0.001) and LC3 (Fig. 1F, H, *p < 0.05) further increased compared to the Mock group. These data suggest that PFV infection induce mitophagy.

### PFV gag induces mitochondrial damage

**Fig. 2:**
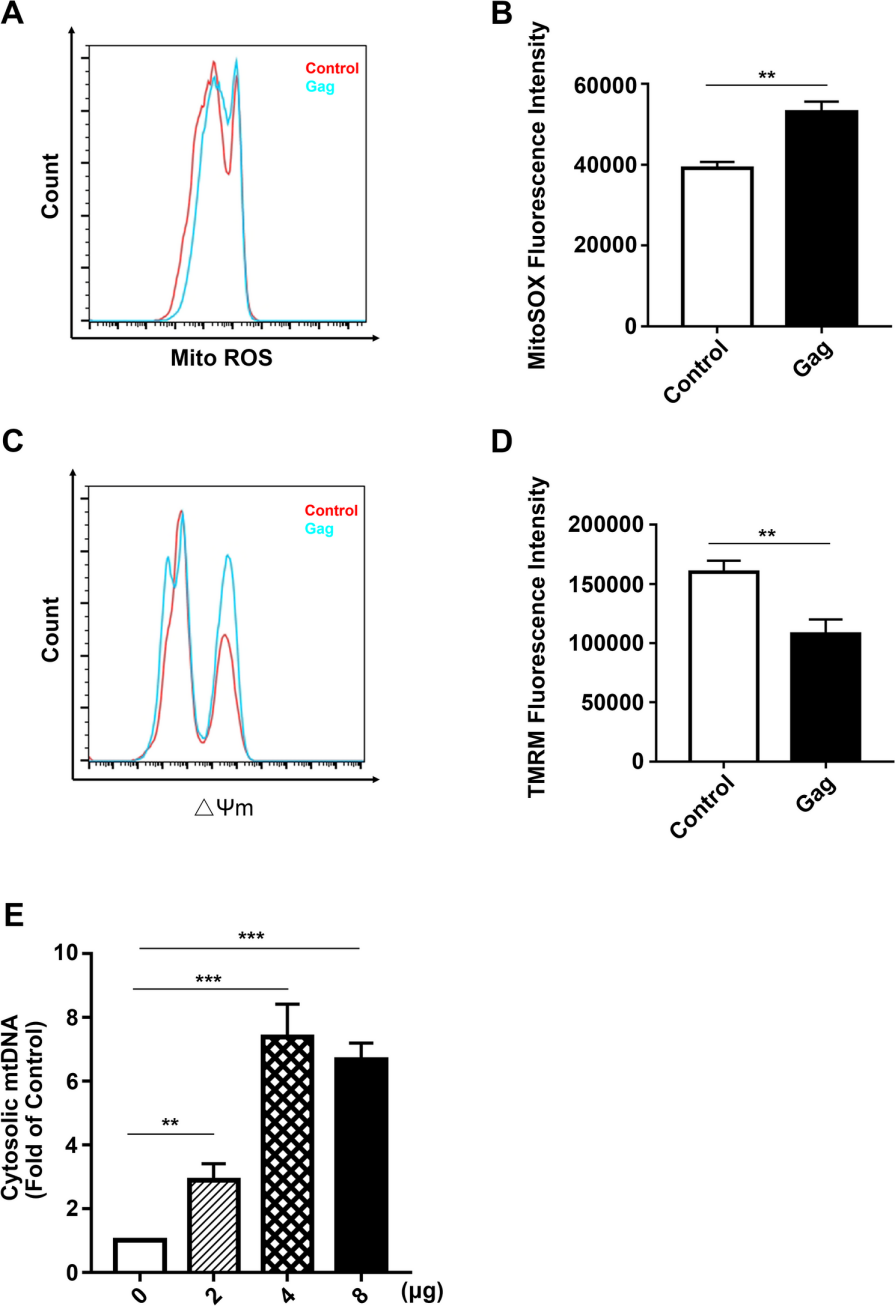
**(A-B)** HEK293T cells were transfected with pCDNA6.0-His-Gag plasmid for 48 h, followed by incubation with 500 nM MitoSOX (protected from light) for 10 min. Changes in fluorescence intensity were quantitatively analyzed using flow cytometry (*n* = 3, **p < 0.01). **(C-D)** The effects of Gag protein on mitochondrial membrane potential were assessed by transfecting HEK293T cells with pCDNA6.0-His-Gag plasmid for 48 h. Cells were then incubated with TMRM at 37 °C for 30 min, washed with PBS, and fluorescence intensity of 200 nM TMRM was quantitatively measured using flow cytometry (*n* = 3, **p < 0.01). **(E)** HEK293T cells were transfected with varying doses (2 µg, 4 µg, 8 µg) of pCDNA6.0-His-Gag plasmid for 48 h. Mitochondrial isolation was performed, and DNA was extracted from the cytoplasm. The content of cytochrome C oxidase I (encoded by mitochondrial DNA) was analyzed as a measure of mitochondrial DNA content, with 18 S rRNA (encoded by nuclear DNA) serving as an internal reference for real-time PCR detection (*n* = 4, **p < 0.01, ***p < 0.001)

Retroviral Gag proteins play a crucial role in regulating the cell membrane system [26]. Our previous studies have found that PFV Gag interacts with Alix to promote endosomal associated autophagy [24], leading us to hypothesize that Gag plays a similar role in mitophagy. First, we investigated whether PFV Gag causes mitochondrial damage. Given that mitochondrial damage leads to mtROS production and the loss of mitochondrial membrane potential (Δψm), we assessed the alterations in mtROS levels and mitochondrial membrane potential following PFV Gag overexpression. In 293T cells with PFV Gag overexpression, we found that mtROS increased significantly and mitochondrial membrane potential decreased significantly compared to control (Fig. 2A-D, **p < 0.01). To further confirm that PFV Gag causes mitochondrial damage, we examined mtDNA release. The result show that PFV Gag promotes mtDNA release into the cytoplasm in a dose-dependent manner (Fig. 2E). In comparison to the control group transfected with empty vector, the release of mtDNA following transfection with 2 µg of the pCDNA6.0-His-Gag plasmid increased by 2.9-fold (Fig. 2E, **p < 0.01). The release of mtDNA after transfection with 4 µg of the pCDNA6.0-His-Gag plasmid increased by 7.4-fold (Fig. 2E, ***p < 0.001), while transfection with 8 µg of the pCDNA6.0-His-Gag plasmid resulted in a 6.7-fold increase in mtDNA release (Fig. 2E, ***p < 0.001). These data suggest that PFV Gag causes mitochondrial damage.

### PFV gag induces mitophagy

Since PFV Gag induces mitochondrial damage, we next explored whether PFV Gag causes mitophagy. Compared with the control group transfected with empty vector, transfection with 400 ng pCDNA6.0-His-Gag plasmid resulted in an average increase of LC3 lipidation by 2-fold and an average decrease of p62 by 0.5-fold (Fig. 3A-C, *p < 0.05). Furthermore, transfection with 800 ng pCDNA6.0-His-Gag plasmid resulted in an average increase of LC3 lipidation by 2.7-fold (Fig. 3A, C, *p < 0.05) and an average decrease of p62 by 0.7 times (Fig. 3A, B, **p < 0.01). PFV Gag promotes LC3 lipidation and p62 degradation in a dose-dependent manner (Fig. 3A-C). Cell isolation analysis showed compared with the control group transfected with empty vector, the mitochondrial-to-cytosolic ratios of p62 and LC3 in cells transfected with 4 µg of the pCDNA6.0-His-Gag plasmid increased by 3.6 times (Fig. 3D, E, **p < 0.01) and 2.2 times (Fig. 3D, F, *p < 0.05) respectively. In cells transfected with 8 µg Gag plasmid, the mitochondrial-to-cytosolic ratios of p62 increased by 11.7 times (Fig. 3D, E, **p < 0.01) and the mitochondrial-to-cytosolic ratios of LC3 increased by 3 times (Fig. 3D, F, **p < 0.01). In conclusion, the mitochondrial-to-cytosolic ratios of p62 and LC3 increased in a dose-dependent manner in cells overexpressed PFV Gag (Fig. 3D-F). This suggests that PFV Gag facilitates the translocation of LC3 and p62 from the cytoplasm to mitochondria.

**Fig. 3-1:**
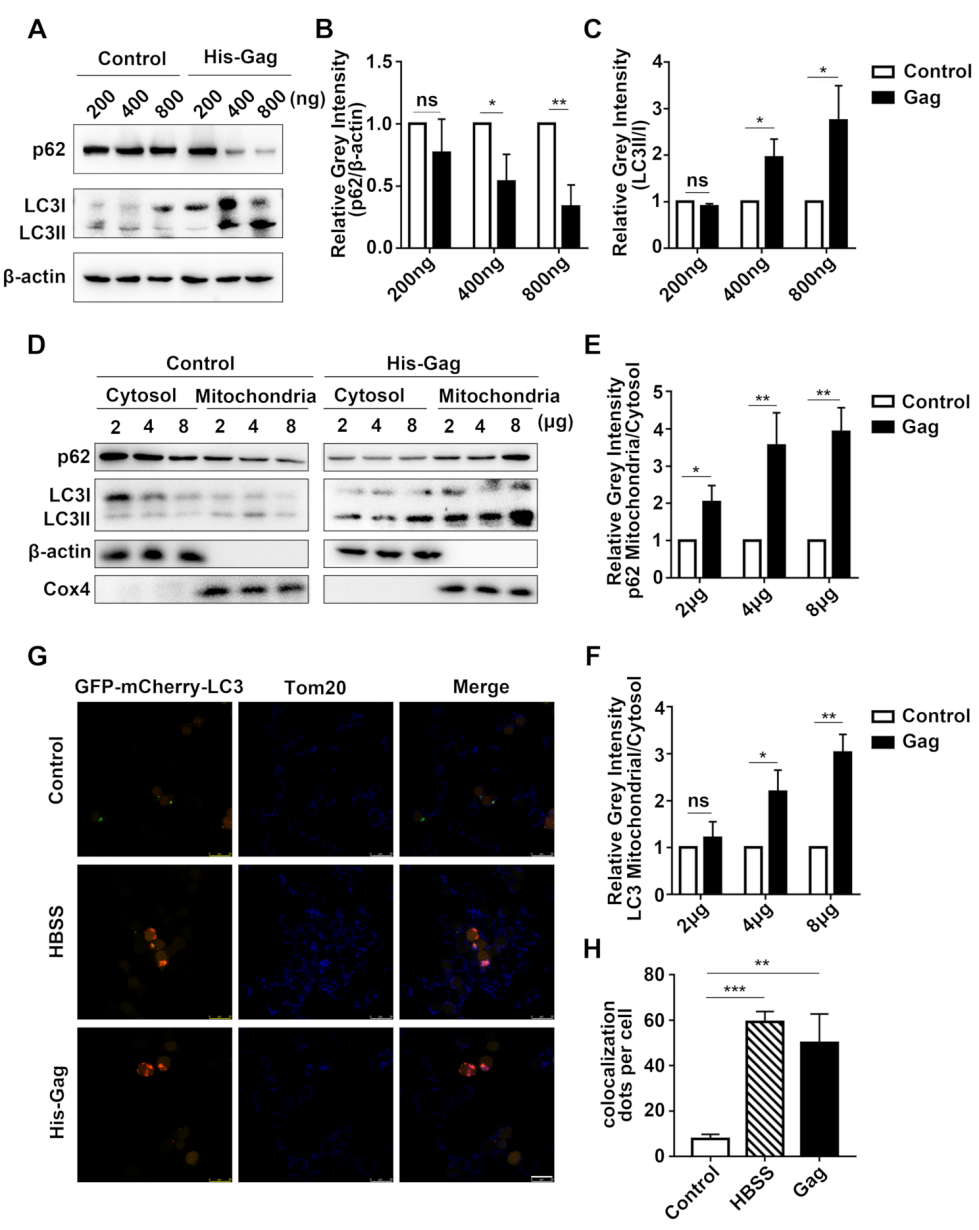
**(A)** HEK293T cells were seeded in 12-well plates and transfected with pcDNA6.0 (empty vector) or pCDNA6.0-His-Gag plasmid at doses of 200 ng, 400 ng, and 800 ng for 48 h. Western blot analysis was performed to assess changes in p62 and LC3 II/I levels. **(B-C)** Statistical analysis of p62 and LC3 II/I protein expression changes (Student’s *t*-test, *n* = 3, *p < 0.05, **p < 0.01, ns *p* > 0.05). **(D)** HEK293T cells (approximately 5 × 10^7^ per dish) were transfected with pcDNA6.0 (empty vector) or pCDNA6.0-His-Gag plasmid (2 µg, 4 µg, 8 µg) for 48 h. Mitochondrial isolation was performed, and Western blot analysis detected changes in p62 and LC3 II/I levels in both cytoplasm and mitochondria. **(E-F)** Statistical analysis of p62 and LC3 II/I protein expression changes (*n* = 3, *p < 0.05, **p < 0.01, ns *p* > 0.05). **(G)** HEK293T cells were transfected with a double fluorescent label plasmid (mCherry-GFP-LC3). After 24 h, cells were divided into three groups: negative control (transfected with empty vector), positive control (starved with HBSS), and experimental group (transfected with pCDNA6.0-His-Gag plasmid). After 48 h, co-localization of LC3 and Tom20 was analyzed using confocal microscopy. Scale bar = 25 μm. **(H)** Statistical analysis of the co-localization of LC3 and Tom20 (Student’s *t*-test, *n* = 30, **p < 0.01, ***p < 0.001)

**Fig. 3-2:**
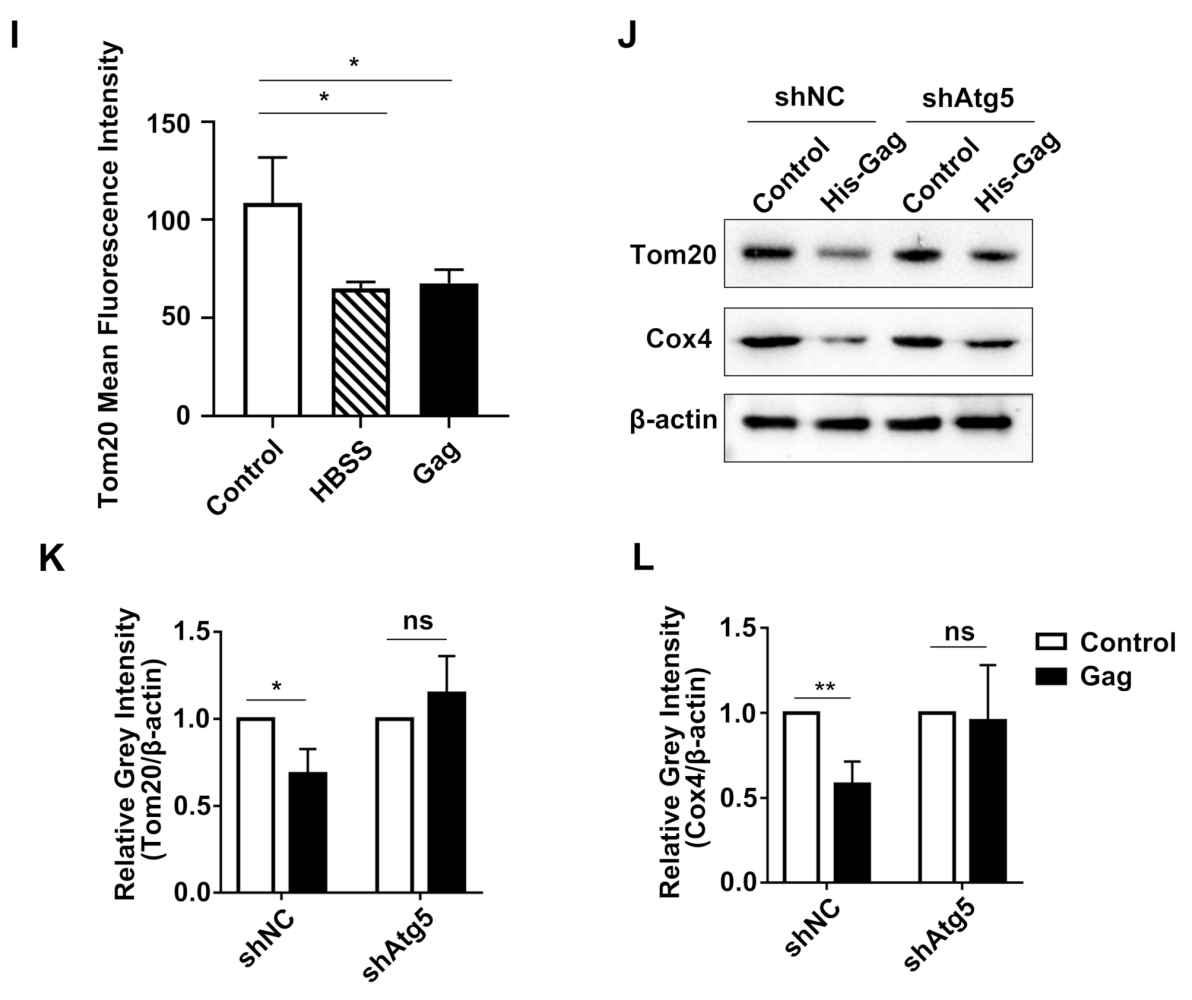
**(I)** Statistical analysis of the fluorescence intensity of Tom20 (Student’s *t*-test, *n* = 30, *p < 0.05) **(J)** After constructing a shAtg5 plasmid to knock down Atg5, HEK293T cells were cotransfected with the shAtg5 and pCDNA6.0-His-Gag plasmid for 48 h. The protein expression levels of Tom20 and Cox4 in the cell lysate were subsequently detected. **(K-L)** Statistical analysis of Tom20 and Cox4 protein expression levels was performed (One-way ANOVA, *n* = 3, *p < 0.05, **p < 0.01, ns *p* > 0.05)

Tom20, a core component of the mitochondrial outer membrane translocase complex, serves as a critical marker for mitophagy activation, as its decreased protein levels directly reflect the initiation of mitochondrial clearance [27]. Next, we used confocal microscopy to detect the co-localization of autophagy marker LC3 and mitochondrial outer membrane protein Tom20 to determine the formation of mitophagosomes. We used Hank’s Balanced Salt Solution (HBSS) treated cells as positive controls and empty vector transfected cells as negative controls. Confocal microscopy confirmed that LC3 and Tom20 co-localization increased in cells overexpressed PFV Gag compared with empty vector transfected cells. (Fig. 3G, H, **p < 0.01). Furthermore, compared with the control group transfected with the empty vector, the fluorescence intensity of Tom20 in the cells transfected with PFV Gag decreased (Fig. 3G, I, *p < 0.05).

Cox4, serving as a subunit of mitochondrial respiratory chain complex IV, is specifically localized to the inner membrane of mitochondria. Its abundance exhibits alterations concomitant with the degradation of mitochondria [28]. Atg5 is essential for autophagosome elongation, and its depletion blocks autophagic flux by preventing LC3 lipidation [29]. To further confirm that PFV Gag induces mitophagy, we detected the levels of mitochondrial proteins Cox4 and Tom20 in PFV Gag overexpressed cells under the condition of Atg5 knockdown. The results showed that both Tom20 (Fig. 3J, K, *p < 0.05) and Cox4 (Fig. 3J, L, **p < 0.01) were reduced in cells that overexpressed PFV Gag, and knocking down Atg5 reversed the reduction of Cox4 and Tom20 caused by PFV Gag to a certain extent (Fig. 3J-L), indicating that knocking down Atg5 inhibited the process of mitophagy. Collectively, these findings indicate that PFV Gag induces mitophagy, a form of mitochondria-specific autophagy.

### PFV gag induces mitophagy in a Parkin-dependent manner

**Fig. 4:**
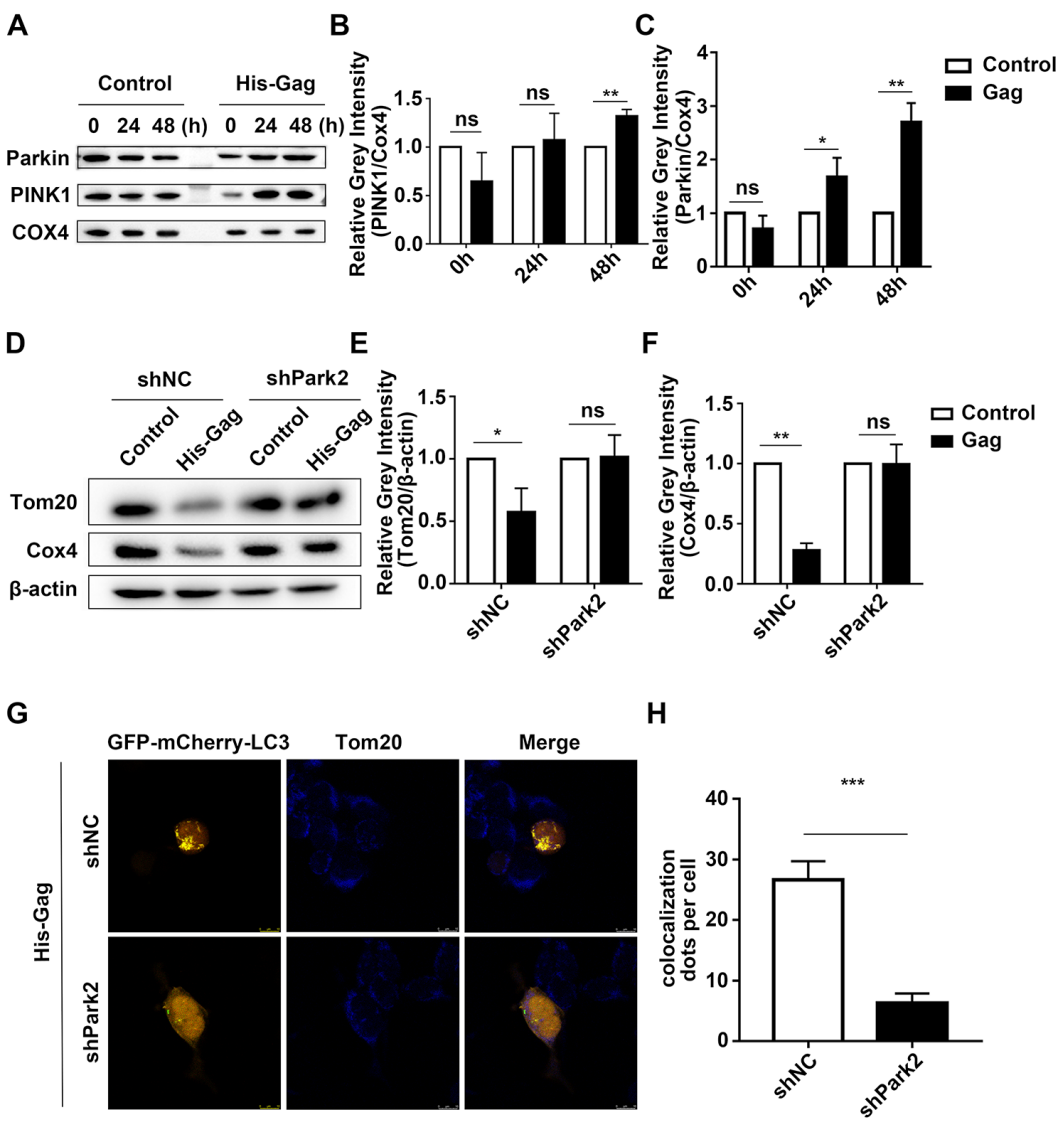
**(A-C)** pCDNA6.0-His-Gag plasmid was transfected into HEK293T cells, and mitochondria were extracted after 48 h. Changes in the protein levels of Parkin and PINK1 in the mitochondria were detected by Western blot and statistically analyzed (*n* = 3, *p < 0.05, **p < 0.01, ns *p* > 0.05). **(D)** shRNA targeting endogenous Parkin was transfected into HEK293T cells for 24 h, followed by transfection with pCDNA6.0-His-Gag plasmid for an additional 48 h. Cell lysates were collected to extract protein, and Western blot analysis was performed to detect changes in Tom20 and Cox4. **(E-F)** Statistical analysis of Tom20 and Cox4 protein expression changes (*n* = 3, *p < 0.05, **p < 0.01, ns *p* > 0.05). **(G)** Following the transfection of shPark2 to knock down endogenous Parkin in HEK293T cells, mCherry-GFP-LC3 and pCDNA6.0-His-Gag plasmid were cotransfected. The co-localization of LC3 and Tom20 was analyzed using confocal microscopy. **(H)** Statistical analysis of the co-localization of LC3 and Tom20. Scale bar = 10 μm. (***p < 0.001)

Furthermore, we explored the mechanism of mitophagy induced by PFV Gag. The expression of Parkin (Fig. 4A, C, **p < 0.01) and PINK1 (Fig. 4A, B, **p < 0.01) is significantly increased in cells overexpressed PFV Gag. To further confirm Parkin is essential for PFV Gag induced mitophagy, the level of mitochondrial proteins Cox4 and Tom20 in PFV Gag overexpressed cells was examined in the condition of Parkin knockdown. After overexpression of PFV Gag, Cox4 (Fig. 4D, F, **p < 0.01) and Tom20 (Fig. 4D, E, *p < 0.05) were reduced, and Parkin knockdown reversed the decrease of Cox4 and Tom20 induced by PFV Gag (Fig. 4D-F). Confocal results showed that the co-localization of LC3 and Tom20 was reduced after Parkin knockdown in PFV Gag overexpressed cells compared to control (Fig. 4G, H, ***p < 0.001). These results suggest that PFV Gag induces Parkin-dependent mitophagy.

### PFV gag promotes Parkin-dependent mitophagy by upregulating Rab5a

**Fig. 5:**
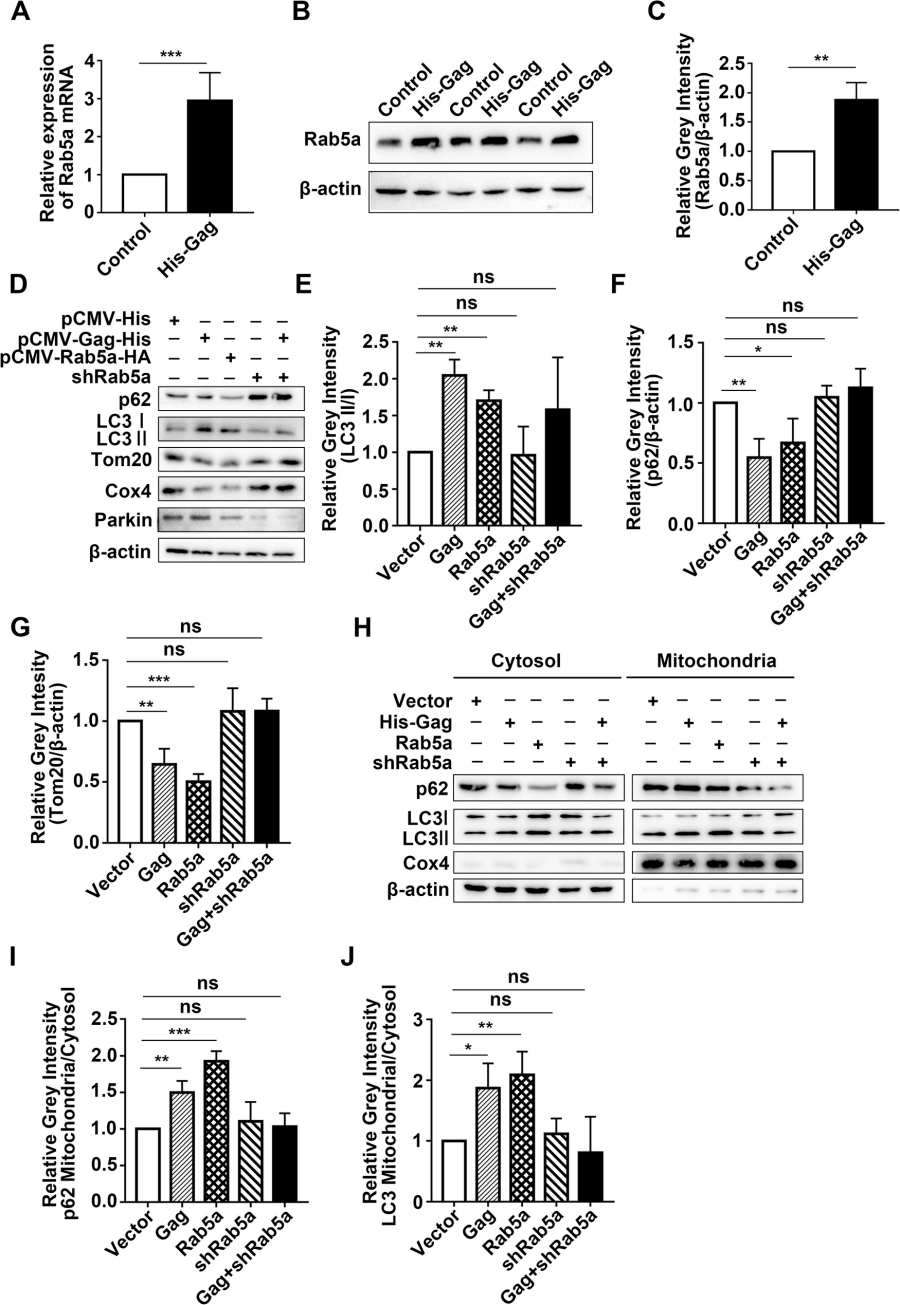
**(A)** After 48 h of transfection with pCDNA6.0-His-Gag plasmid in HEK293T cells, real-time PCR was performed to detect changes in Rab5a mRNA levels. **(B**,** C)** Western blot analysis revealed changes in Rab5a protein levels following 48 h of transfection with pCDNA6.0-His-Gag plasmid in HEK293T cells (**p < 0.01, ***p < 0.001). **(D)** HEK293T cells were transfected with pcDNA6.0 (empty vector), pCDNA6.0-His-Gag plasmid, Rab5a expression plasmid, shRNA targeting Rab5a, or cotransfected with pCDNA6.0-His-Gag plasmid and shRNA targeting Rab5a. Cell lysates were collected, proteins extracted, and Western blotting was used to detect changes in p62, LC3 II/I, and Tom20. **(E-G)** Statistical analysis of p62, Tom20, and LC3 II/I protein levels (*n* = 3, *p < 0.05, **p < 0.01, ***p < 0.001, ns *p* > 0.05). **(H)** Following the same transfection as in **(D)**, cells were collected for mitochondrial isolation. Proteins in mitochondria were extracted, and Western blot analysis was performed to assess changes in p62 and LC3 II/I in cytoplasm and mitochondria. **(I-J)** Statistical analysis of changes in p62 and LC3 II/I in cytoplasmic mitochondria. (Student’s *t*-test, *n* = 3, *p < 0.05, **p < 0.01, ***p < 0.001, ns *p* > 0.05)

Many Rab proteins have been reported to be involved in the formation of autophagosomes [30]. Among them, Rab5a is involved in a variety of antiviral immune reactions and is essential for early endosome formation [31]. It is interesting to determine whether Gag affects the expression of Rab5a. Both mRNA (Fig. 5A, ***p < 0.001) and protein levels (Fig. 5B, C, **p < 0.01) of Rab5a were increased in cells overexpressed PFV Gag. Subsequently, we investigated the role of Rab5a in PFV Gag induced mitophagy. Our results indicated that the overexpression of PFV Gag or Rab5a led to the degradation of p62, increased lipidation of LC3, and a reduction in Tom20 levels compared to the control group. Furthermore, knocking down Rab5a was found to reverse the PFV Gag induced degradation of p62, lipidation of LC3, and decrease in Tom20 (Fig. 5D-G). Cell isolation analysis demonstrated that, in comparison to the control group transfected with empty vector, the accumulation of p62 (Fig. 5H, I, **p < 0.01) and LC3 (Fig. 5H, J, *p < 0.05) on mitochondria was significantly higher in cells overexpressed PFV Gag. Similarly, cells overexpressed Rab5a also exhibited increased levels of p62 (Fig. 5H, I, ***p < 0.001) and LC3 (Fig. 5H, J, **p < 0.01) in mitochondria. Importantly, the knockdown of Rab5a negated the PFV Gag induced increases in LC3 and p62 levels within the mitochondria. (Fig. 5H-J). These results indicate that PFV Gag promotes mitophagy by upregulating Rab5a.

## Discussion

Here we report that PFV induces mitochondrial damage and triggers mitophagy in infected host cells. In addition, Gag protein is a key molecule in PFV-induced mitophagy and can induce Parkin-dependent mitophagy by upregulating Rab5a. These results suggested that PFV might utilize mitophagy to promote its own survival.

Previous work demonstrated that foamy virus infection triggers endoplasmic reticulum stress [23] and endosome-related autophagy. Specifically, PFV Gag promotes the formation of amphisomes, which helps clear stress granules and inhibits type I interferon responses [24]. Mitophagy is crucial for maintaining mitochondrial health and function. By removing damaged or aged mitochondria, cells prevent oxidative stress and inflammation, thus preserving intracellular homeostasis. In this paper, mitophagy induced by foamy virus infection may significantly influence the viral replication cycle and may affect host cell metabolism and immune responses.

Mitochondria play an important role in viral immunity. Viruses can exploit host mitophagy pathways to evade antiviral immunity. Some viruses induce mitophagy through Parkin-independent mechanisms. The ORF10 protein of SARS-CoV-2 interacts with the mitophagy receptor NIX, leading to its translocation to the mitochondria. The NIX-LC3 interaction promotes mitophagy, resulting in MAVS degradation and suppression of the type I interferon response [32]. The PB2-F1 protein of influenza A virus interacts with the autophagy receptor LC3 via its LIR domain, driving mitophagy and MAVS degradation to suppress the innate immune response [33]. Similarly, the matrix protein (M) of human parainfluenza virus type 3 (HPIV3) induces mitophagy by interacting with TUFM and LC3, promoting mitophagosome formation [34]. Conversely, some viruses induce Parkin-dependent mitophagy. Retroviruses like HIV-1 exemplify Parkin-dependent mechanisms, where infection of central nervous system microglia activates the NLRP3 inflammasome. This activation drives mitochondrial ROS overproduction, triggering PINK1 stabilization and subsequent Parkin recruitment to depolarized mitochondria, ultimately leading to mitophagic clearance of MAVS-containing mitochondria and attenuation of type I interferon signaling [35]. In this study, we found that the retrovirus PFV induces Parkin-dependent mitophagy through its structural protein Gag. Moreover, we observed a reduction in IFNB secretion (Supplementary Fig. 1A, ***p < 0.001) and decreased mRNA levels of IFNB (Supplementary Fig. 1B, **p < 0.01), CXCL5 (Supplementary Fig. 1C, ***p < 0.001), and CXCL10 (Supplementary Fig. 1D, **p < 0.01) following PFV Gag overexpression. This suggests that PFV Gag may suppress the innate immune response of host cells. However, the precise mechanisms by which PFV Gag induces mitophagy via Rab5a and its impact on the host cell’s innate immune response require further investigation.

Rab5a, a small GTPase protein from the Rab family, is crucial for early endosomal biosynthesis [36]. Our previous research indicated that the host protein TBC1D16 inhibits PFV replication by regulating Rab5C [37]. Additionally, some studies have highlighted the role of the Rab5 endosomal pathway in mediating Parkin-dependent mitochondrial clearance [38]. In our current study, we observed a significant increase in the mRNA level and protein level of Rab5a following PFV Gag overexpression, suggesting that Rab5a might be involved in PFV Gag-induced mitophagy. Further investigation revealed that PFV Gag induces Parkin-dependent mitophagy by upregulating Rab5a. This represents the first report of a retrovirus using its Gag protein to induce Parkin-dependent mitophagy through the upregulation of Rab5a. This finding not only uncovers a novel connection between foamy virus infection and host cell mitochondrial quality control mechanisms but also provides a theoretical basis for developing new therapeutic strategies and interventions against foamy virus infections. Furthermore, it offers valuable insights for the development of antiviral strategies targeting other retroviruses and for addressing viral diseases more broadly, thereby presenting important scientific and clinical prospects.

## Conclusion

**Fig. 6:**
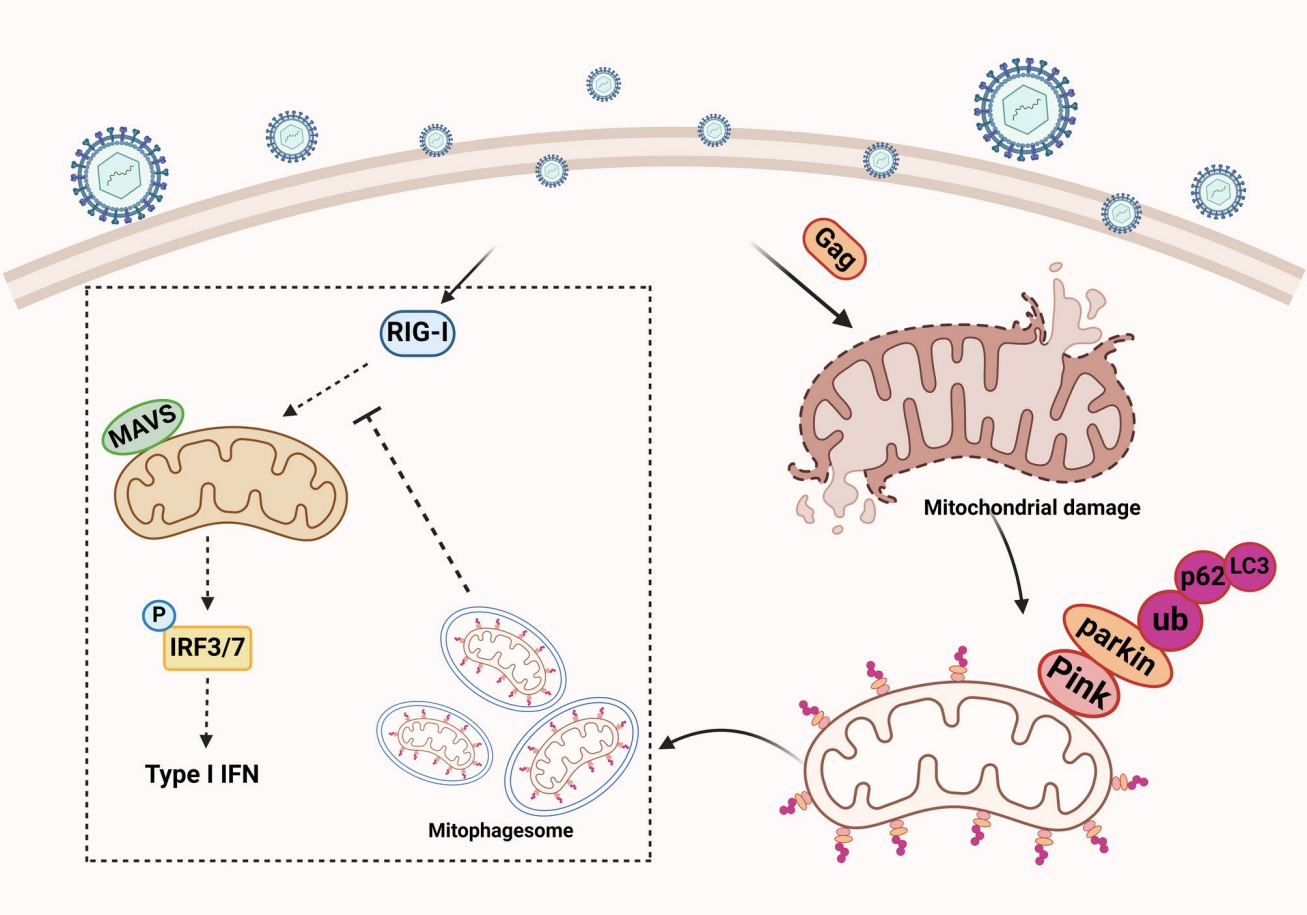
Schematic diagram of mitophagy induced by PFV

In summary (Fig. 6), We demonstrated that PFV can cause mitochondrial damage and induce mitophagy following the infection of host cells. Our findings indicate that PFV Gag is a novel structural protein that triggers mitophagy and serves as a key molecule in PFV-induced mitophagy. PFV Gag promotes Parkin-dependent mitophagy via Rab5a. Additionally, we discovered that PFV Gag can inhibit the type I interferon response in host cells, potentially through its role in mitophagy.

## Electronic supplementary material

Below is the link to the electronic supplementary material.

Supplementary Material 1: Fig. 1. (A) ELISA detects changes in IFNB secretion. (B-D) qPCR analysis reveals alterations in mRNA levels of IFNB, CCL5, and CXCL10 following Gag protein overexpression. (*n* = 3, **p < 0.01, ***p < 0.001).

Supplementary Material 2

## Data Availability

No datasets were generated or analysed during the current study.

## Abbreviations

PFV: Prototype foamy virus
mtROS: Mitochondria reactive oxygen species
SFV: Simian foamy virus
PINK1: PTEN-induced putative kinase 1
NIX: NIP3-like protein X
FUNDC1: FUN14 domain-containing protein 1
BNIP3: Bcl-2/adenovirus E1B 19 kDa interacting protein 3
HBV: Hepatitis B virus
HCV: Hepatitis C virus
VEEV: Venezuelan equine encephalitis virus
CSFV: Classical swine fever virus
PRRSV: Porcine reproductive and respiratory syndrome virus
NDV: Newcastle disease virus
TGEV: Transmissible gastroenteritis virus
HPIV3: Human parainfluenza virus type 3
HIV-1: Human immunodeficiency virus type 1
IFNB: Interferon-beta
MOI: Multiplicity of infection
