# Supplementary figures and images for: Retroviral foamy virus gag induces parkin-dependent mitophagy
Source: Retrovirology. 2025 May 2;22:7. doi: 10.1186/s12977-025-00664-3 (PMC12048983; doi:10.1186/s12977-025-00664-3)

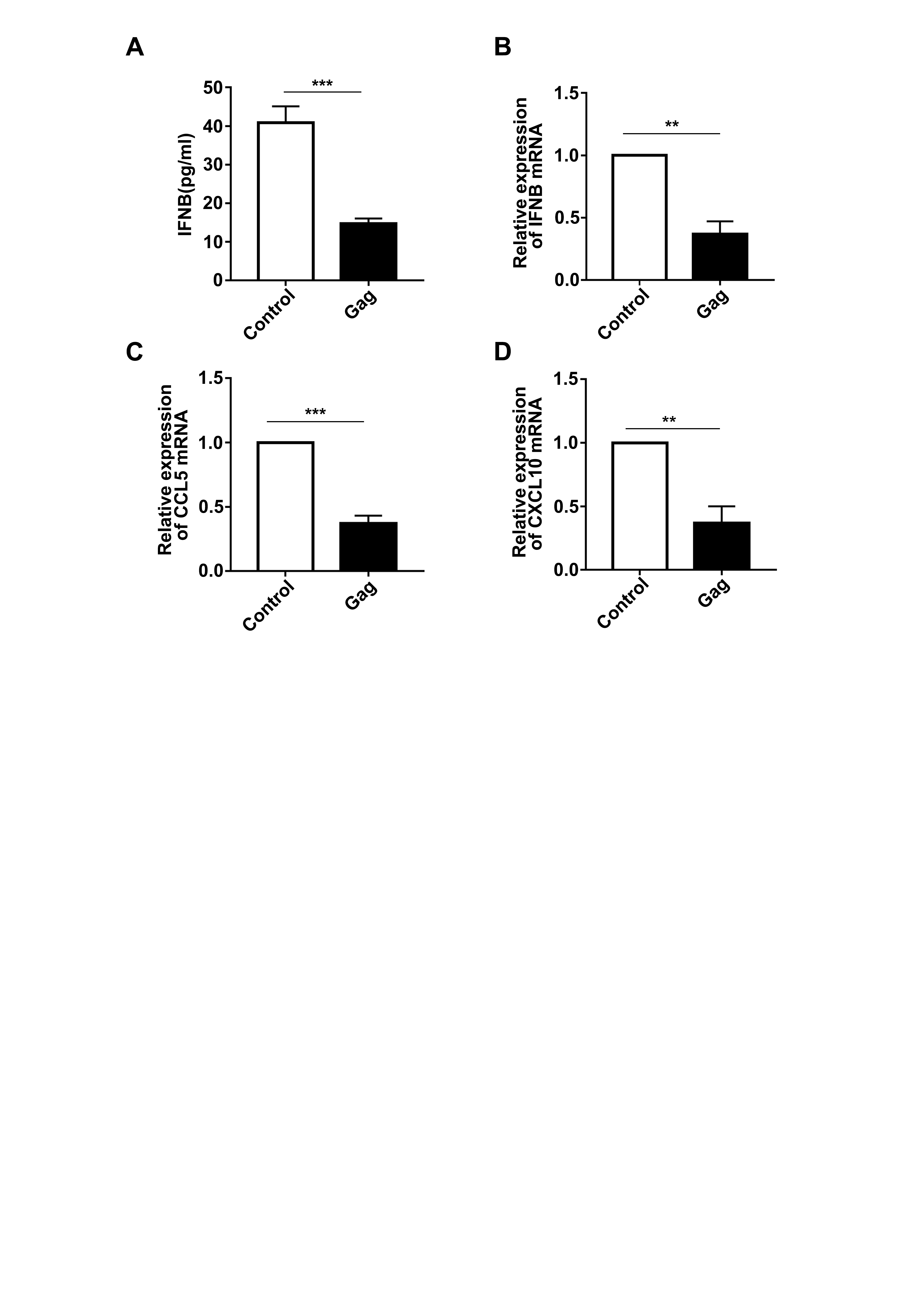

Supplement: Supplementary file 1 — Supplementary Material 1: Fig. 1. (A) ELISA detects changes in IFNB secretion. (B-D) qPCR analysis reveals alterations in mRNA levels of IFNB, CCL5, and CXCL10 following Gag protein overexpression. (n = 3, **p < 0.01, ***p < 0.001). [file 12977_2025_664_MOESM1_ESM.tif]
