# Supplementary material for: Retroviral foamy virus gag induces parkin-dependent mitophagy
Source: Retrovirology. 2025 May 2;22:7. doi: 10.1186/s12977-025-00664-3 (PMC12048983; doi:10.1186/s12977-025-00664-3)
Supplement: Supplementary file 2 — Supplementary Material 2 [file 12977_2025_664_MOESM2_ESM.pptx]

## Slide 1
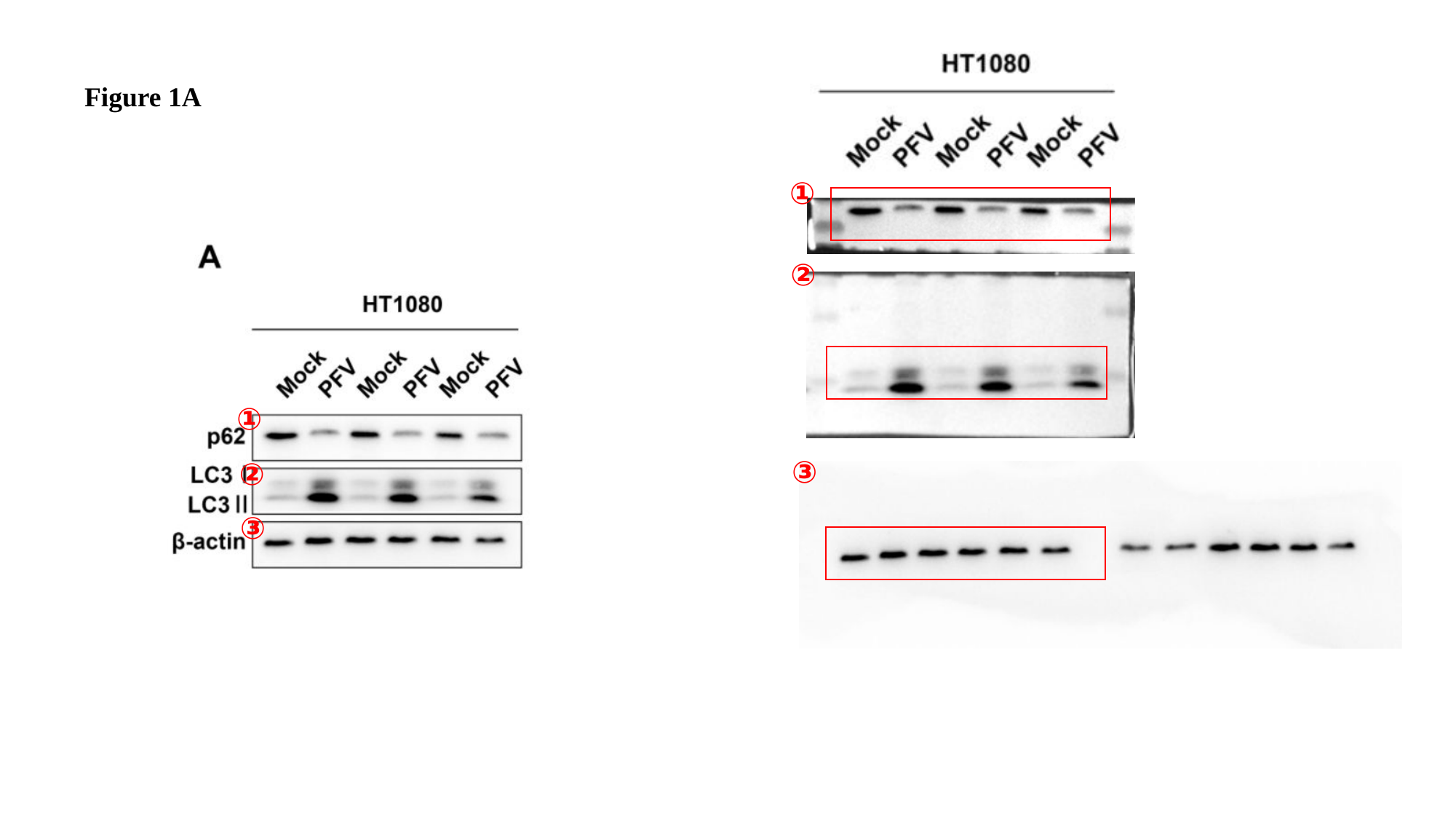

Figure 1A
①
②
①
③
②
③

## Slide 2
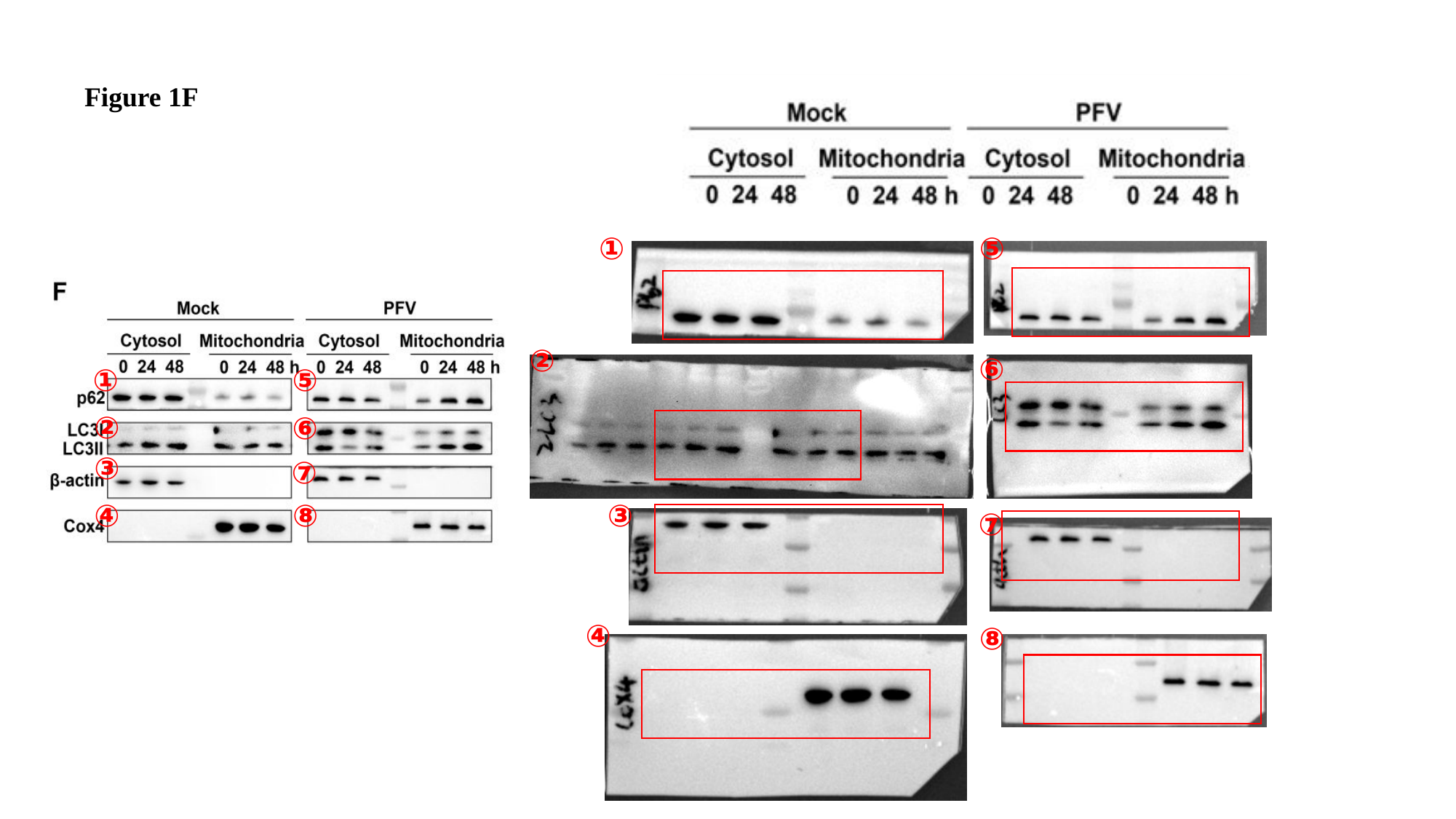

Figure 1F
⑤
①
②
⑥
①
⑤
②
⑥
③
⑦
④
⑧
③
⑦
④
⑧

## Slide 3
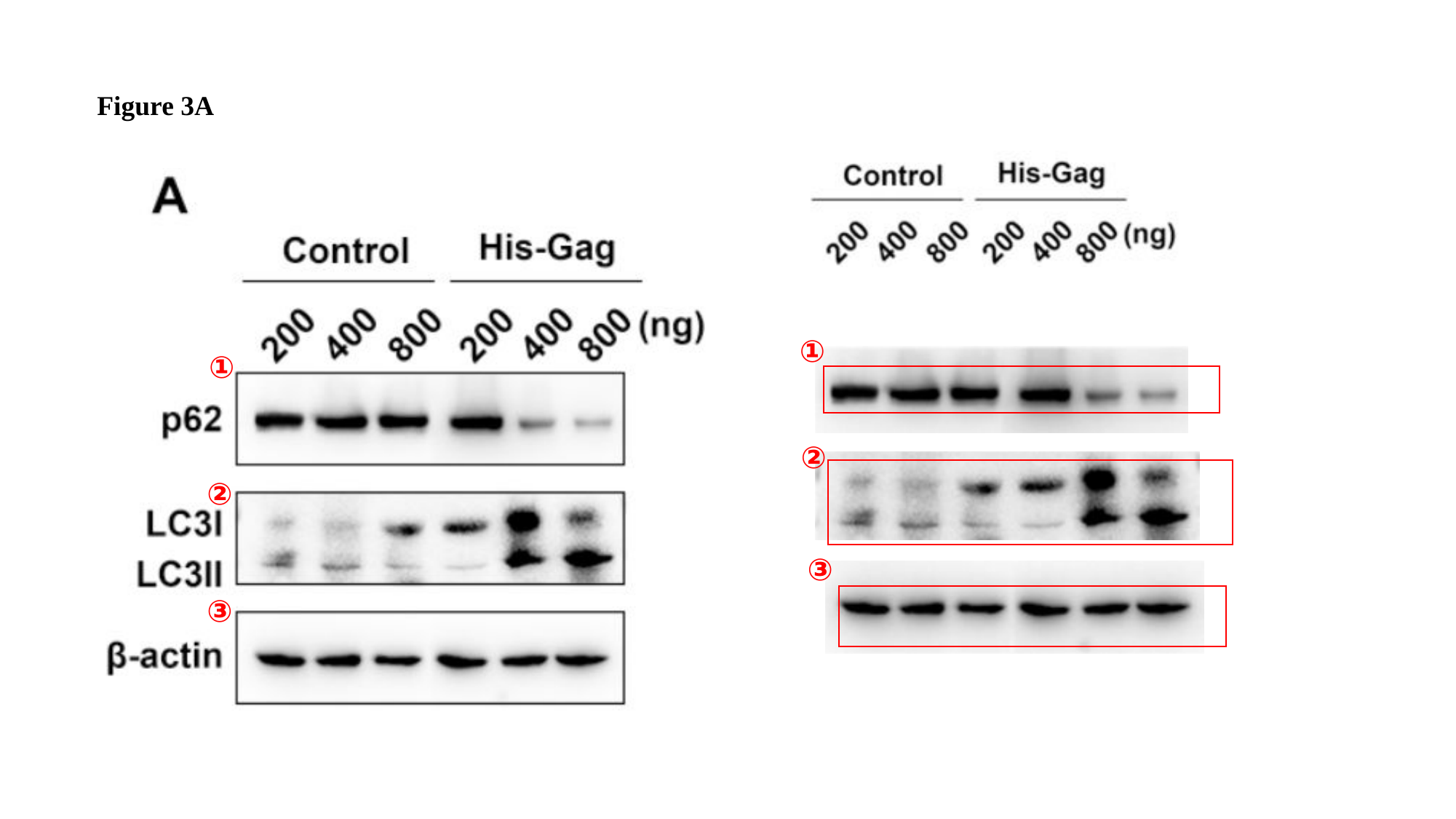

Figure 3A
①
①
②
②
③
③

## Slide 4
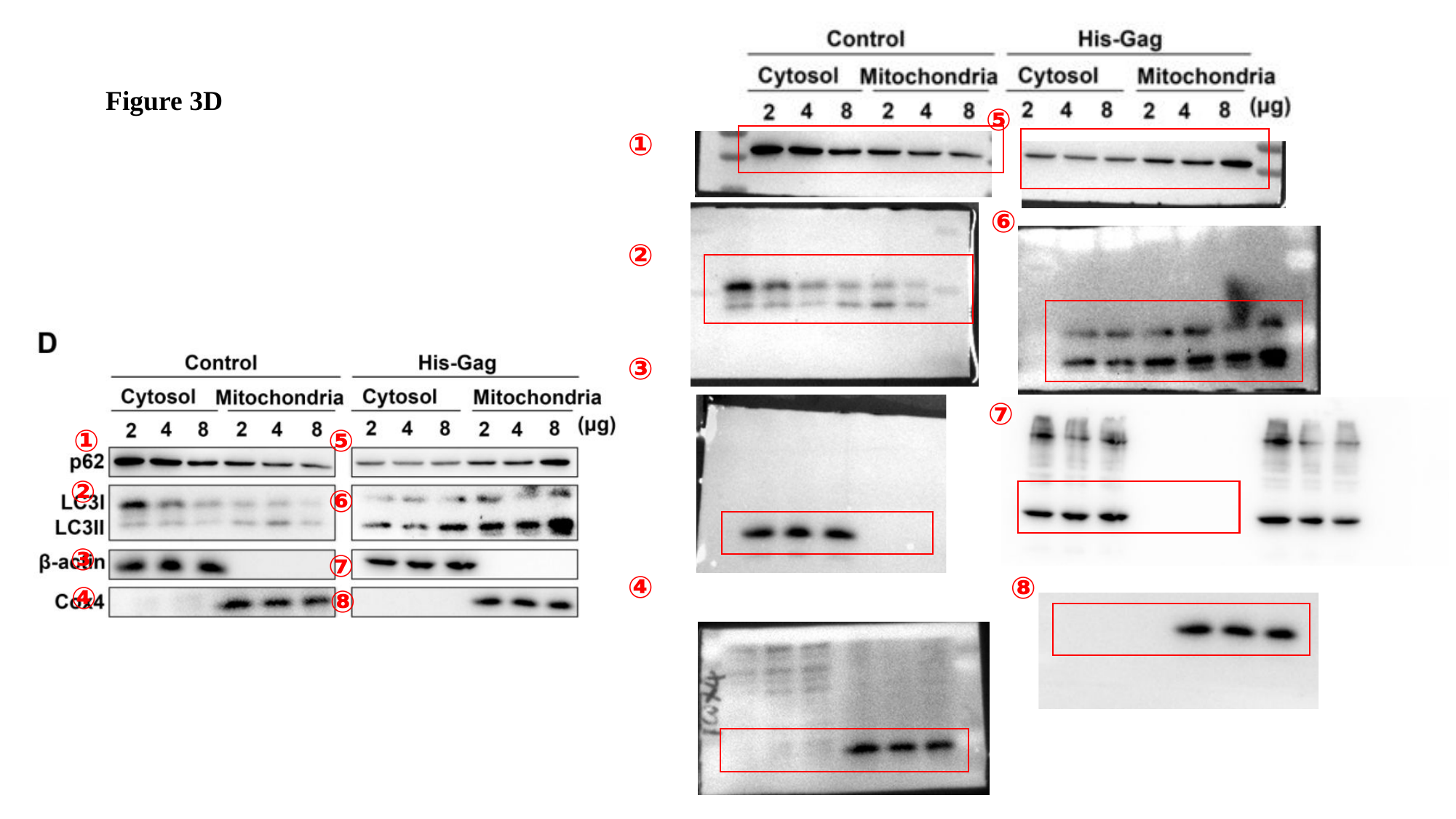

Figure 3D
⑤
①
⑥
②
③
⑦
①
⑤
②
⑥
③
⑦
④
⑧
④
⑧

## Slide 5
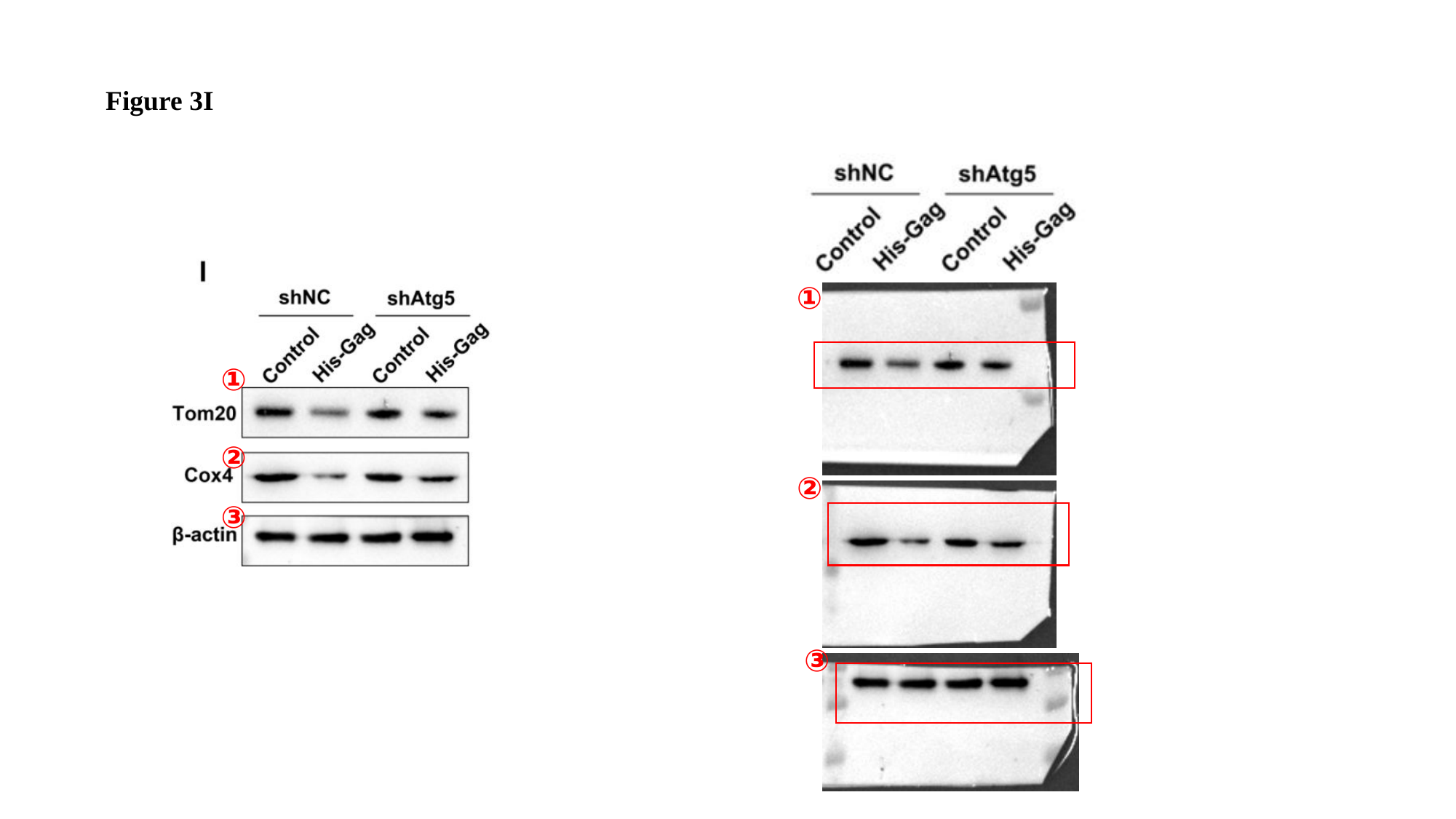

Figure 3I
①
①
②
②
③
③

## Slide 6
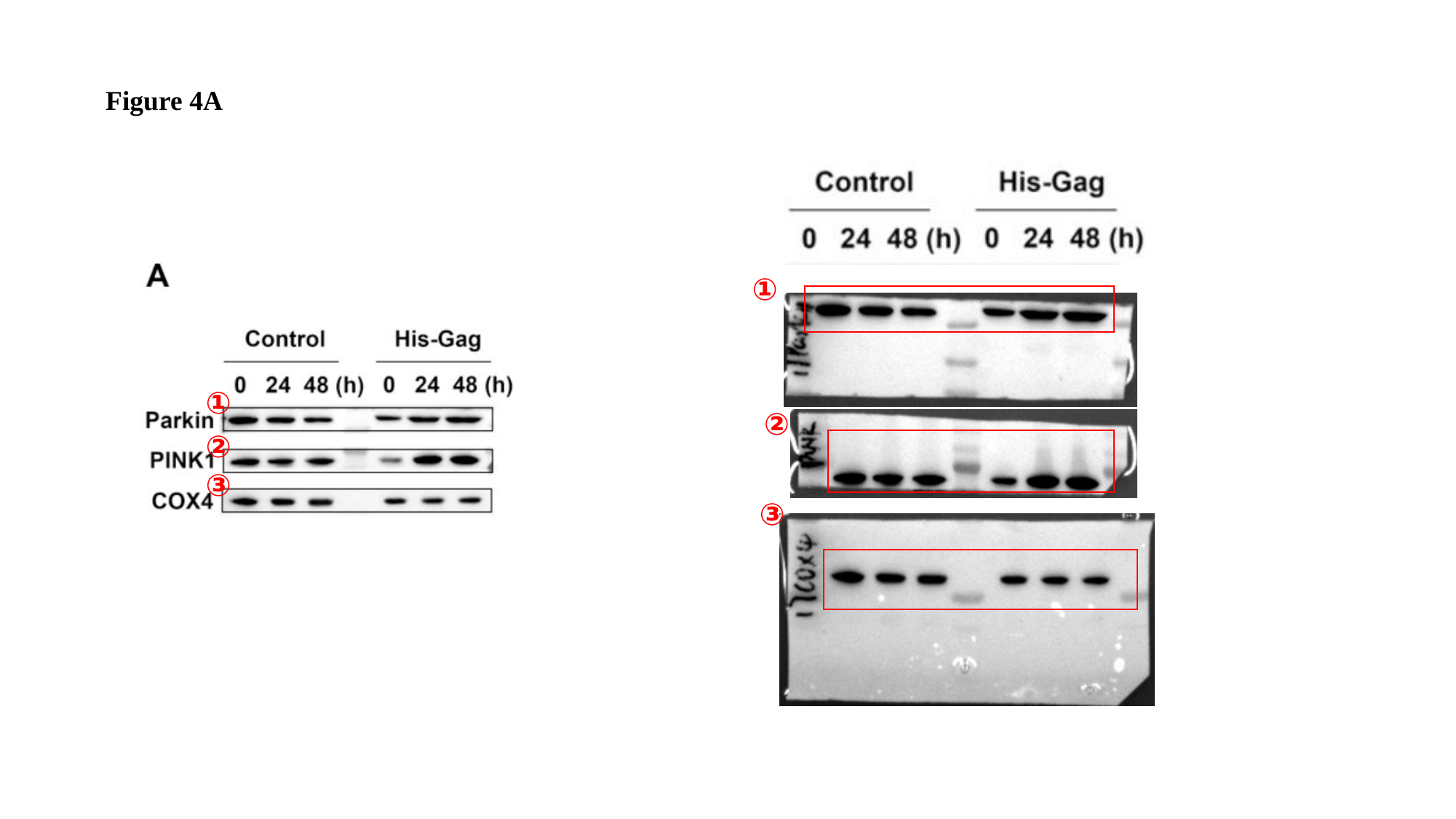

Figure 4A
①
①
②
②
③
③

## Slide 7
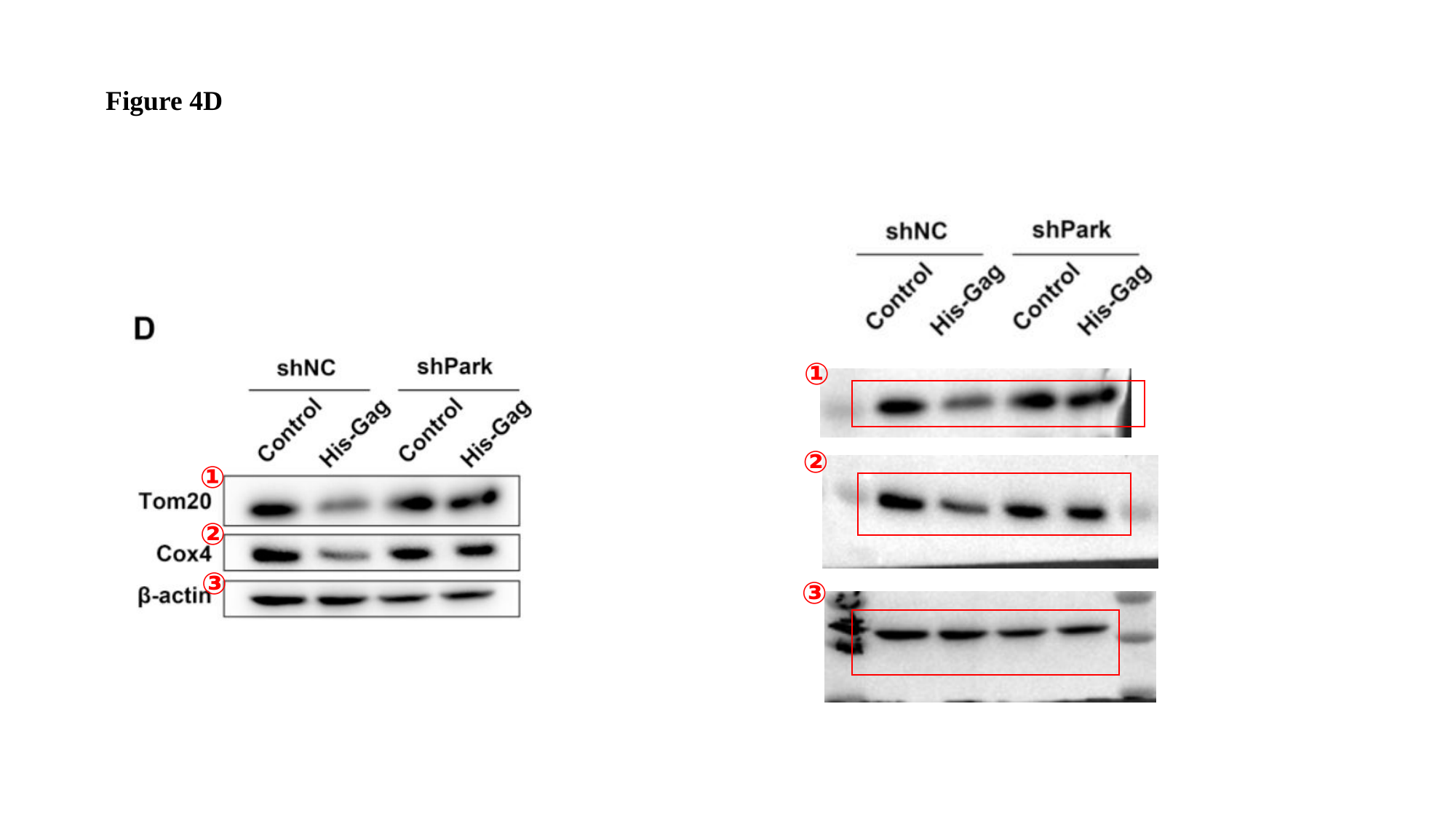

Figure 4D
①
②
①
②
③
③

## Slide 8
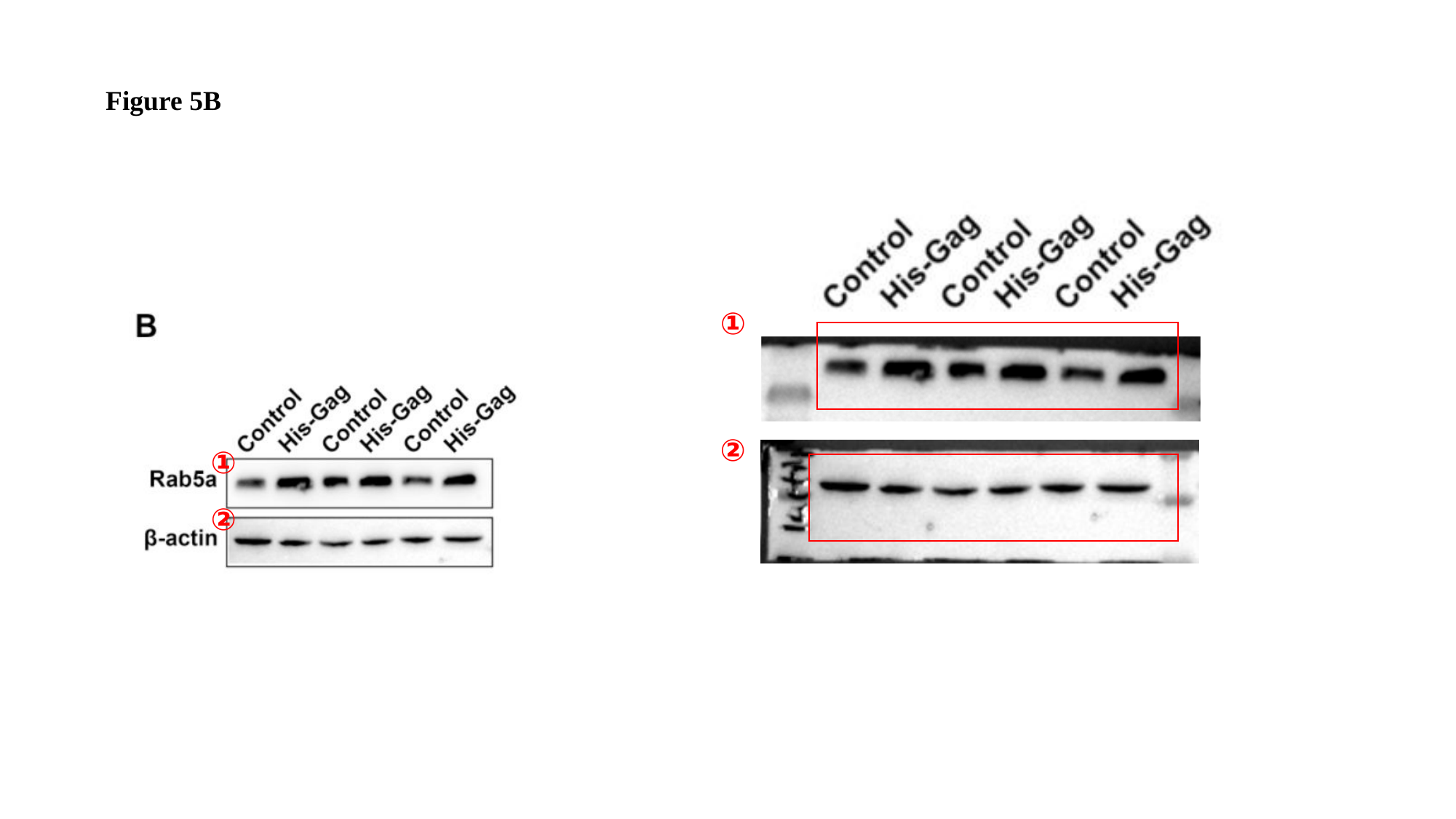

Figure 5B
①
②
①
②

## Slide 9
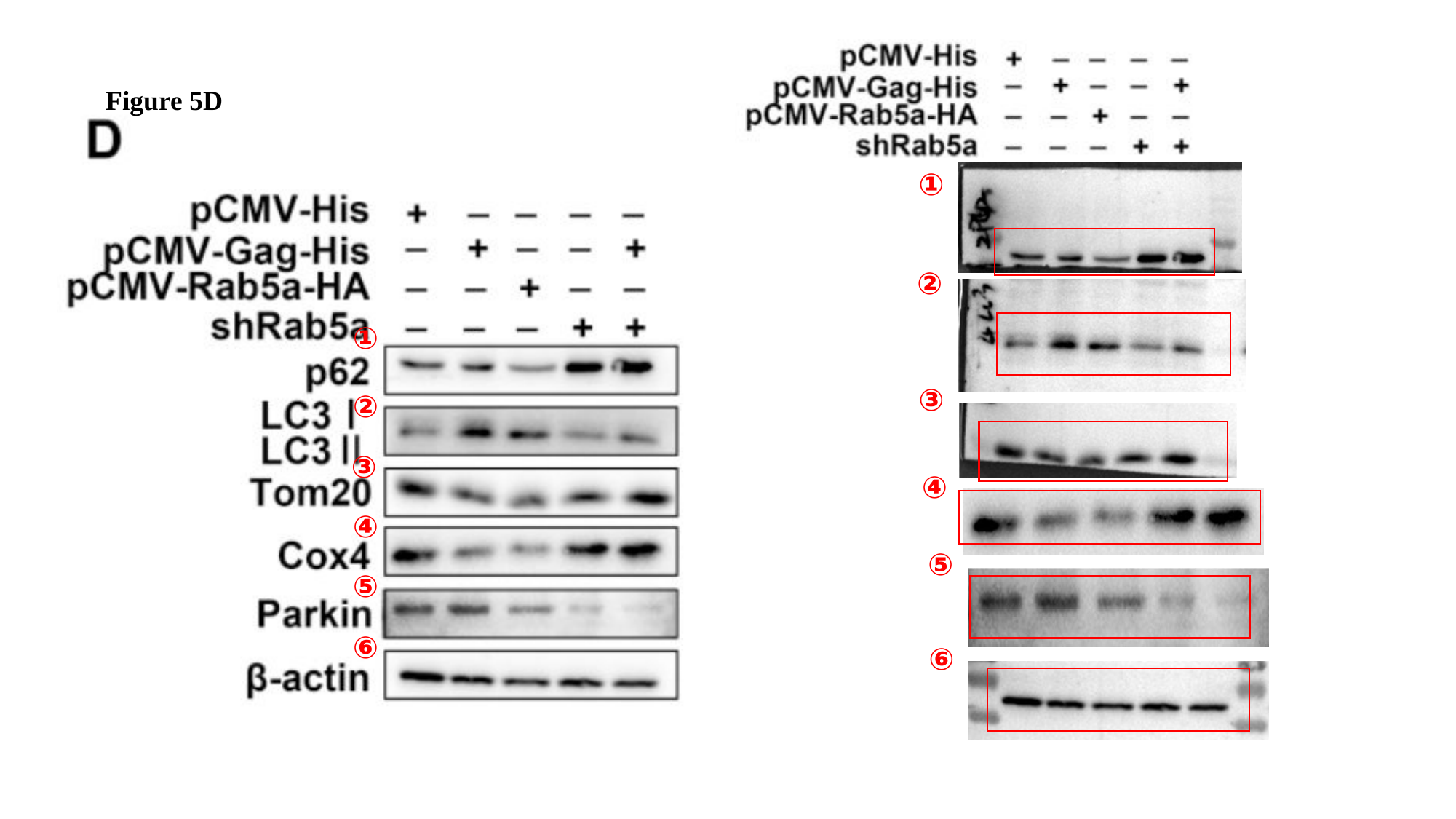

Figure 5D
①
②
①
③
②
③
④
④
⑤
⑤
⑥
⑥

## Slide 10
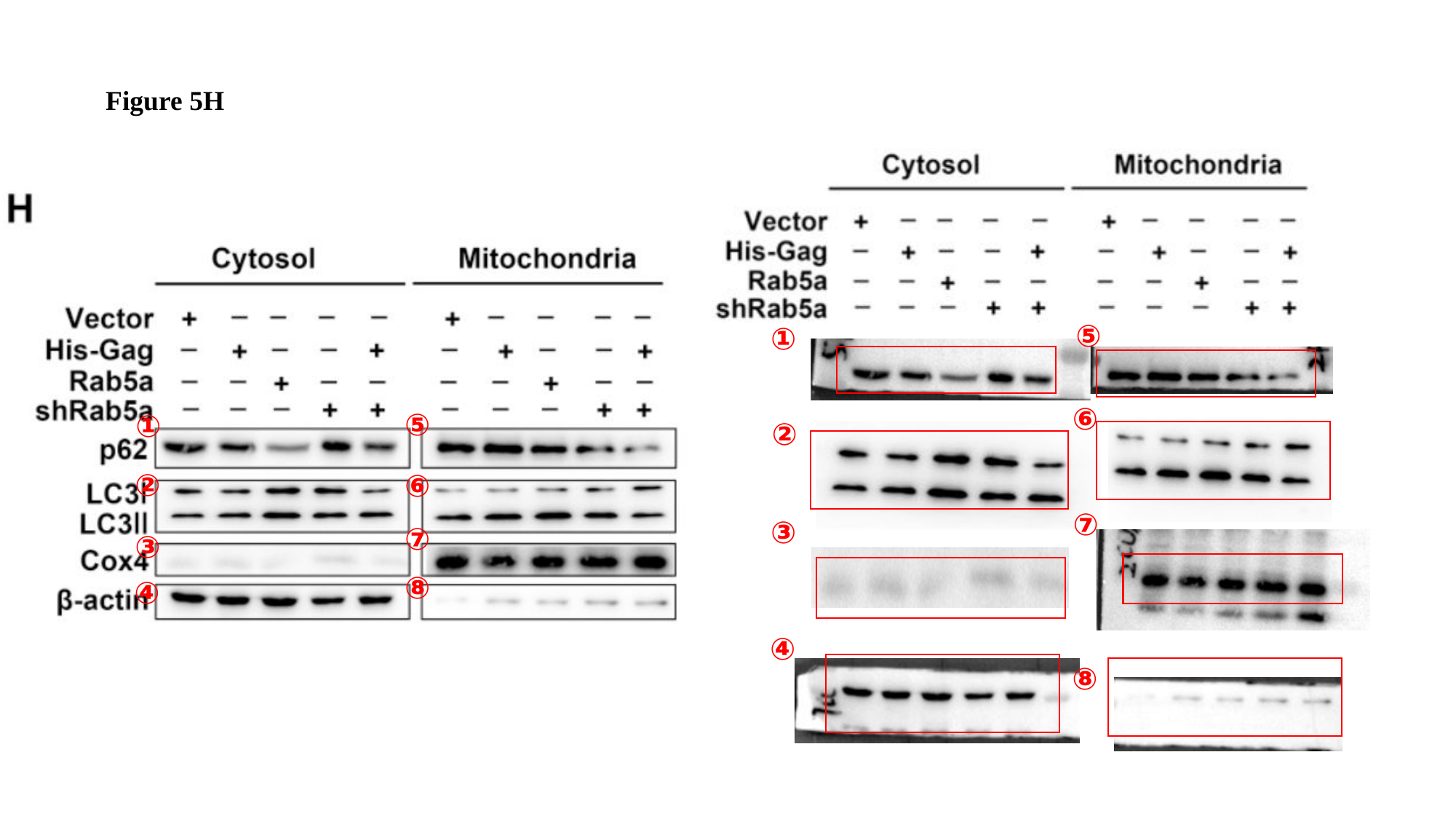

Figure 5H
⑤
①
⑥
⑤
①
②
②
⑥
⑦
③
⑦
③
⑧
④
④
⑧
